# Nanoencapsulation of Cyanidin 3-*O*-Glucoside: Purpose, Technique, Bioavailability, and Stability

**DOI:** 10.3390/nano13030617

**Published:** 2023-02-03

**Authors:** Oscar Zannou, Kouame F. Oussou, Ifagbémi B. Chabi, Nour M. H. Awad, Midimahu V. Aïssi, Gulden Goksen, Mustafa Mortas, Fatih Oz, Charalampos Proestos, Adéchola P. P. Kayodé

**Affiliations:** 1 Department of Food Engineering, Faculty of Engineering, Ondokuz Mayis University, 55139 Samsun, Turkey; 2 Laboratory of Human Nutrition and Valorization of Food Bio-Ingredients, Faculty of Agricultural Sciences, University of Abomey-Calavi, Cotonou 01 BP 526, Benin; 3 Department of Food Engineering, Faculty of Agriculture, Çukurova University, 01330 Adana, Turkey; 4 School of Sciences and Techniques for the Conservation and Processing of Agricultural Products, National University of Agriculture, Sakété 00 BP 144, Benin; 5 Department of Food Technology, Vocational School of Technical Sciences at Mersin Tarsus Organized Industrial Zone, Tarsus University, 33100 Mersin, Turkey; 6 Department of Food Engineering, Agriculture Faculty, Atatürk University, 25240 Erzurum, Turkey; 7 Food Chemistry Laboratory, Department of Chemistry, National and Kapodistrian University of Athens, Panepistimiopolis Zographou, 15771 Athens, Greece

**Keywords:** cyanidin 3-*O*-glucoside, extraction, nanoencapsulation, nanocarriers, bioavailability

## Abstract

The current growing attractiveness of natural dyes around the world is a consequence of the increasing rejection of synthetic dyes whose use is increasingly criticized. The great interest in natural pigments from herbal origin such as cyanidin 3-*O*-glucoside (C3G) is due to their biological properties and their health benefits. However, the chemical instability of C3G during processing and storage and its low bioavailability limits its food application. Nanoencapsulation technology using appropriate nanocarriers is revolutionizing the use of anthocyanin, including C3G. Owing to the chemical stability and functional benefits that this new nanotechnology provides to the latter, its industrial application is now extending to the pharmaceutical and cosmetic fields. This review focuses on the various nanoencapsulation techniques used and the chemical and biological benefits induced to C3G.

## 1. Introduction

Cyanidin 3-*O*-glucoside (C3G) is a secondary metabolite naturally present in plants. It is a water-soluble bioactive compound belonging to the class of monomeric anthocyanin, one of the main classes of the flavonoid family. Recently, there has been considerable interest in work on anthocyanin derivatives. There are two main reasons for this increased interest: (i) anthocyanin derivatives are important sources of natural pigments primarily used as food colorants, (ii) and their regular consumption has significant health benefits. They are extensively used in coloring various food products such as cheese, biscuits, yogurt, porridge, and others [1,2,3]. As in the food field, these natural pigments have found their application in the pharmaceutical and cosmetic fields as a substitute for synthetic dyes [3], which are more and more criticized owing to their high toxicity [4]. Without being a panacea, natural colorants, particularly C3G, now appear as a healthy and promising alternative to synthetic dyes, and their application fields continue to grow year after year. Despite these functional attributes, C3G is poorly bioavailable and its absorption in the small intestine is very limited, which affects its functionality in vivo [4,5,6]. However, the major shortcoming of this class of bioactive compound is its instability. Anthocyanin, especially C3G, is known to be very sensitive to pH variation, oxygen, light, and elevated temperature, resulting in its degradation during food processing. For example, C3G oxidation releases unwanted compounds that are responsible for the development of an undesirable taste and off-odor in foods [7,8]. Thus, C3G’s stability and it’s in vivo bioavailability have become a matter of concern not only for the scientific community but also for many industrial applications.

In the last few years, several studies have focused on techniques and methods to ensure C3G’s stability during food processing and storage, as well as improving its bioavailability both in vitro and in vivo [4,5,9]. Among the many explored techniques, the recently developed nanoencapsulation technology is at the forefront. This new technology consists in enclosing an active molecule in gaseous, liquid, or solid state within a matrix to form a nanometric-sized capsule. Encapsulation technology is widely applied in the food and pharmaceutical fields to encapsulate bioactive molecules by creating a protective wall against their degradation factors, such as light, oxygen, pH, moisture, temperature, and others [10,11]. In this technology, the matrix plays an extremely pivotal role since it preserves the coated active molecule and increases its stability. Gum Arabic, xanthan gum, maltodextrin, and alginate are part of the successful nanocarriers reported in the literature for anthocyanin nanoencapsulation [5,11]. According to Shishir et al. [11], in addition to the above-mentioned role, encapsulation technology improves the bioavailability and bio-accessibility of active molecules and enhances their functionality. Recently, some overviews have focused on C3G, studying its physical properties, chemistry [1], health effects [1,2,3], and its mechanism of maintaining intestinal integrity [4]; however, no overviews have underlined the nanoencapsulation of C3G. The current review article aims at highlighting C3G extraction methods, the nanoencapsulation techniques, as well as the nanocarriers used for its encapsulation. An overview of the effects of these techniques on the chemical stability, bioavailability, and especially on the functionality of C3G has been performed.

## 2. Chemical Structure and Health Properties of Cyanidin 3-*O*-Glucoside

**Figure 1:**
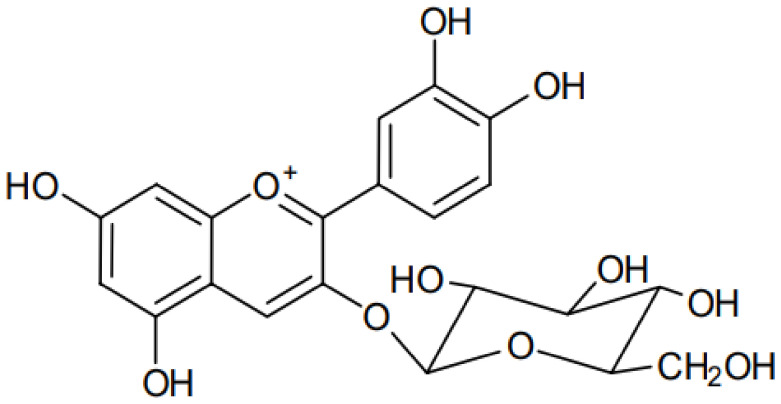
Chemical structure of cyanidin 3-*O*-glucoside.

C3G (Figure 1) is an anthocyanin compound derived from the aglycone cyanidin. It is a red pigment of anthocyanin commonly and naturally found in vegetables and fruits. C3G is highly hydrophilic, with a solubility of 0.6 mg/mL, LogP of 0.39, Å^2^ of 193.4, [M]+ (*m*/*z*) of 449, and MS/MS (*m*/*z*) of 287 [12].

The UV-vis spectrum of C3G at different pH (pH 3–8) revealed that C3G is pH-dependent [13]. In this case, it was found that the flavylium cation absorbance is dominant at pH 3, whose intensity decreases at pH 4–5, but at pH 5, the quinoidal species starts dominating. At pH 6–8, cyandin-3-glucoside takes the quinoidal and chalcone forms.

C3G is a well-known natural anthocyanin and possesses tremendous health benefits (Table 1). Wang et al. [14] studied the ability of C3G and its phenolic acid metabolites to attenuate the visible light-induced retinal degeneration and found out that the C3G, protocatechuic acid, and ferulic acid caused a higher secretion and expression of heme oxygenase (HO-1) and nuclear factor erythroid-2-related factor 2 (Nrf2), indicating the attenuation of the secretion or expression of inflammation-related genes. Likewise, the treatments attenuated the photo-oxidation-induced apoptosis and angiogenesis in the retina. Du et al. [15] reported that the C3G as well as cyanidin chloride hinder the accumulation of cholesterol and inhibit the LXRα pathway-induced inflammatory response. In the study of Chen et al. [16], it was found that the C3G and peonidin 3-glucoside exhibited anticancer activity via G2/M arrest with peonidin 3-glucoside treatment, showing downregulation of protein levels of cyclin-dependent kinase (CDK)-1, CDK-2, cyclin B1, and cyclin E, while cyanidin 3-*O*-glucoside reduced the protein levels of CDK-1, CDK-2, cyclin B1, and cyclin D1. The potency of C3G to improve the hyperglycemia and insulin sensitivity in diabetic mice fed with dietary C3G for 5 weeks was investigated [17]. The results indicated that C3G has a great antidiabetic ability via regulation of the Glut4-RBP4 system and inflammatory adipocytokines. Ziberna et al. [18] demonstrated that the C3G could be transported into endothelial cells via bilitranslocase and provided powerful intracellular antioxidant and cardioprotective effects in endothelial cells. In a recent study, Tan et al. [19] evaluated the effect of binding C3G and blueberry pectin on antioxidant activity. They showed that pectin wrapped the C3G via a hydrogen bond interaction, leading to an increase of antioxidant activity. In addition, the administration of a diet containing ∼8 mg/kg/day to the rats with signs of metabolic syndrome, including visceral adiposity, impaired glucose tolerance, hypertension, cardiovascular remodeling, increased collagen deposition in the left ventricle, non-alcoholic fatty liver disease, increased plasma liver enzymes, and increased inflammatory cell infiltration in the heart and liver, reversed all these metabolic syndrome signs [20]. Furthermore, the administration of C3G to the rats with a carbon tetrachloride (CCl_4_)-induced liver injury was revealed to improve the state of liver-injured rats via antioxidant and anti-inflammatory mechanisms [21]. Yu et al. [22] confirmed that on one hand, C3G attenuated H_2_O_2_-induced cell apoptosis via ROS scavenging, and on the other hand, it has a hepatoprotective effect, alleviating CCl4-induced liver damage in mice. Moreover, C3G has displayed anti-obesity activity in mice subjected to high-fat and high-fructose diets [23].

## 3. Extraction of Cyanidin 3-*O*-Glucoside

The recovery of anthocyanins is affected by the extraction techniques and extraction conditions, such as temperature, type of solvents, solvent ratio, and extraction time [24,25]. The influence of extraction techniques, type of solvents, and raw materials are shown in Table 2. Conventional techniques such as Soxhlet extraction and maceration are widely used for extraction of phenolic compounds such as C3G. For example, Santos et al. [26] successfully applied the Soxhlet extraction to recover C3G from Jabuticaba (*Myrciaria cauliflora*) skins. Likewise, Ćujić et al. [27] and Meregalli et al. [28] determined that the maceration extraction can be used to extract the C3G from various plant materials. Nonetheless, considering these studies on the extraction of C3G by conventional techniques, it can be evidenced that they are time-consuming and mainly depend on the type of solvent. The emerging extraction technologies proved to be more efficient regarding the extraction yield of C3G. A comparative study implying ultrasound-assisted extraction, agitated bed extraction, combined ultrasound-assisted extraction + agitated bed extraction, and Soxhlet extraction has been carried out for the extraction of C3G, and the results showed that the use of shorter ultrasonic irradiation is viable and increased the extraction performance [26]. Although the use of ultrasound-assisted extraction can imply high energy consumption, it is more efficient, rapid, and selective regarding the extraction of anthocyanin than the conventional extraction techniques such as agitated bed extraction, maceration, and Soxhlet extraction [29,30].

Microwave-assisted extraction, which uses the energy of microwaves to induce the molecular movement and rotation of liquids with a permanent dipole, is also an emerging extraction technology for the recovery of C3G [31]. The implementation of microwave-assisted extraction causes, through the energy of microwaves, a rapid heating of the solvent and the sample, leading to the release of bioactive compounds. Microwave-assisted extraction has been found highly efficient for the extraction of anthocyanin derivatives such as C3G, pelargonidin 3-glucoside, and peonidin 3-glucoside from purple corn when compared to the conventional extraction procedure [32]. Zou et al. [33] proved that the microwave-assisted extraction performed at the optimum conditions of 59.6% acidified methanol, 425 W of power, a 25 (*v*/*w*) liquid-to-solid ratio, and a time of 132 s, is efficient for the recovery of C3G from mulberry, while Yang and Zhai [32] proposed the optimum extraction conditions of 19 min, a 1:20 solid:liquid ratio, and a 555 W microwave irradiation power for the recovery of anthocyanin from purple corn.

Ohmic heating technology is also based on the application of electric fields, and use of its thermal nature allows for a controllable heating rate, unrestricted treatment time scale, unrestricted type of waveform and frequency, and the presence of alternating moderate to low electric fields [34]. It is generally used as a pre-treatment prior to the extraction to increase the efficiency of the extraction technique. The grape skins have been pre-treated by ohmic heating, followed by aqueous extraction under a gentle orbital shaking system [34]. It resulted that ohmic heating allowed to boost extraction levels of total phenolic compounds, delphinidin 3-*O*-glucoside, C3G, petunidin 3-glucoside, peonidin 3-*O*-glucoside-3-glucoside, and malvidin 3-*O*-glucoside, as well as the conductivity, soluble solids, and red color intensity of the obtained extracts. The application of ohmic heating allows not only the increase of extraction yield but also reduces the extraction time [35].

High hydrostatic pressure is a cold pasteurization method used as a good alternative to heat-based treatments, consisting of subjecting samples to pressures up to 1000 MPa inside a vessel filled mainly with water, acting as a pressure-transmitting medium. During the high hydrostatic pressure processing, the pressure is transferred into the sample in an isostatic and quasi-instantaneous manner [36]. Recently, a comparative study of ultrasound-assisted, microwave-assisted, high hydrostatic pressure, and conventional extraction methods has been conducted for the extraction of C3G, and the results showed that the high hydrostatic pressure extraction technique can be used for the recovery of C3G, although ultrasound- and microwave-assisted extractions are more efficient [36]. The application of high hydrostatic pressure extraction (600 MPa/5 min/25 °C) as a pre-treatment prior to the extraction of anthocyanin from açai pulps resulted in the increase of the extraction yield by 37% on average [37].

Supercritical CO_2_ extraction uses carbon dioxide above its critical point and exhibits liquid- and gas-like proprieties. Supercritical and subcritical CO_2_ extractions have been commonly used to improve the extraction and selectivity of plant bioactive compounds. Babova et al. [38] applied the supercritical and subcritical CO_2_ extractions to maximize the recovery of anthocyanin from bilberry. These techniques have selectively and highly extracted C3G, as well as cyanidin 3-*O*-arabinoside, delphinidin 3-*O*-glucoside, ellagic acid pentoside, feruloyl hexoside, and quercetin glycosides. Moreover, the use of co-solvents such as water and ethanol in the supercritical CO_2_ extraction increases the recovery C3G [39]. Pressurized fluid extraction is a nonthermal technique that exposes the fluid to pressures above 6 bar and temperatures above 100 °C in the case of water [40]. Tamkutė et al. [41] recovered anthocyanins from the ethanolic extracts of the defatted cranberry pomace obtained by pressurized fluid extraction (83 °C at 103 bar for 3 extraction cycles of 15 min each), and the concentration of C3G was found in ranges of 7.27 ± 0.06–15.09 ± 0.14 mg/100 g. Pressurized liquid extraction was evaluated at 10 MPa and 40, 60, and 80 °C for the extraction of anthocyanin from juçara (*Euterpe edulis* Mart.) using ethanol, water, an acidified mixture of ethanol + water, and acidified water as solvents. A range of 0.0026–0.204 mg/g was achieved for C3G, and the highest anthocyanin content was achieved with acidified water at 40 °C [42]. In a recent study, the maximum extraction of anthocyanins (cyanidin 3-galactoside, cyanidin 3-arabinoside, peonidin 3-galactoside, and peonidin 3-arabinoside) from cranberry pomace was achieved within pressurized ethanol (100%) at 40–120 °C/50 bar [40]. As can be evidenced, the type of solvent and the identification methods also influence the recovery yield of cyandin-3-glucoside.

## 4. Different Nanocarriers of Cyanidin 3-*O*-Glucoside

**Figure 2:**
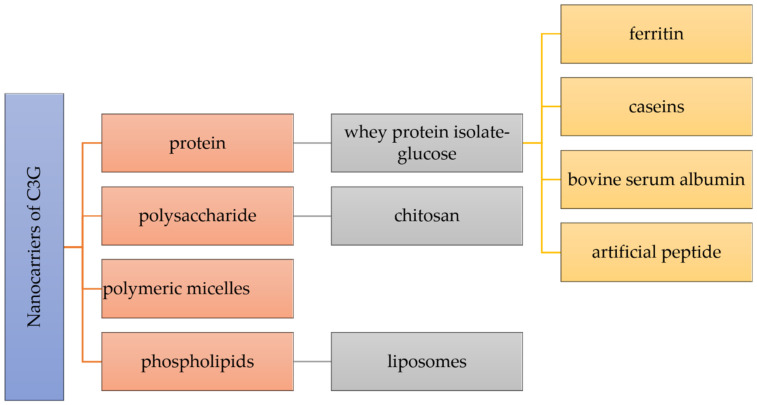
Different nanocarriers used for the encapsulation of cyanidin 3-*O*-glucoside.

The stability, controlled release, intestinal absorption, and bioavailability of nutraceuticals can be improved by a key strategy depending on the creation of nanocarriers. As preferable carrying materials, proteins, polysaccharides, and phospholipids obtained from food are biocompatible, biodegradable, and “generally recognized as safe” (Figure 2) [6].

Many types of nanocarriers have been utilized for the delivery of C3G, including whey protein isolate-glucose (WPI-Glu) nanoparticles, which demonstrated a unique protein delivery method that provides high stability of C3G in acidic and high-temperature conditions [43]. C3G molecules were encapsulated within a ferritin nanocage in order to increase their stability [44]. By encapsulating nanotechnology, protein cage designs such as ferritins provide a strong potential to increase the stability of C3G. The first protein with a shell-like structure employed for the creation of nanomaterials is ferritin, which is a particular kind of widely distributed iron-storage protein [44]. The molecule ferritin, which has a molecular mass of about 500 kDa and external and internal diameters of 12 and 8 nm, is made up of 24 subunits that self-assemble into a shell-like structure with 432 symmetries. Ferritin is used to oxidize and store iron as microcrystalline, hydrated ferric oxide particles. The protein shell of ferritin has a remarkable resistance to chemical and physical denaturants, and the reassembly technique might be utilized to encapsulate such unstable small species, such as C3G, that could not enter the protein cavity through the subunit junction pores [44]. Therefore, C3G was encapsulated in a ferritin nanocage using the reassembling ability of ferritin. It was discovered that such encapsulation significantly improved anthocyanin stability while facilitating C3G transport through Caco-2 cell monolayers [44]. Another protein that has been used as a nanocarrier of C3G is α-casein [45]. Caseins are the major proteins in bovine milk (approximately 80% of the total milk proteins). In the research, the authors assessed the impact of various casein concentrations on the thermal and oxidation stability of C3G and they concluded that caseins may be employed as a natural nano-delivery vehicle and that they formed complexes with C3G by hydrogen bonding and hydrophobic reactions, successfully preventing C3G from deterioration and enhancing the thermal, oxidation, and photostability properties [45]. Similarly, a study examined the stability of C3G when encapsulated with bovine serum albumin to produce beverages [46]. On the other hand, in another published study, C3G has been encapsulated via an intermolecular co-assembly mechanism with an artificial peptide, named C6M1 (RLWRLLWRLWRRLWRLLR), for examining how this interaction between anthocyanin and the peptide affects the stability of C3G [47].

However, because of the poor water solubility, poor bioavailability, and structural instability, the application is still difficult [48]. Sun et al. have evaluated the application of β-cyclodextrin-epichlorohydrin-grafted carboxymethyl chitosan nanocarriers for improving the stability of C3G through the formation of well-dispersed nanoparticles. The nanocomposites of β-CD and carboxymethyl chitosan (CMC), which combine the properties of β-CD and CMC, are a unique encapsulating method for delivering bioactive constituents. The β-CD-EP-CMC nanoparticles used in this investigation had a nearly spherical shape, excellent dispersion, and homogenous morphology [49]. β-cyclodextrin (β-CD) is a commercial agent applied in the food industry with low toxicity. It is a chemically and physically stable molecule, formed by the enzymatic modification of starch, and the enzyme cyclodextrin glucosyl-transferases (CGTs) degrade starch through an intramolecular chain-splitting reaction, producing cyclodextrins [50]. They have the capacity to combine with a wide range of organic substances to create inclusion complexes, which can completely or partially occupy the cavity of CDs, which is generally hydrophobic. The size of the β-CD cavity enables the selective complexation of guest molecules [51]. By enhancing the dissolution rate, membrane permeability, and bioavailability of low-solubility nutraceuticals, CD encapsulation has the potential to significantly alter the physical, chemical, and biological aspects of the molecules it contains. In addition to functioning as flavor carriers, CDs also serve as a barrier against oxidation, light-induced decomposition, and heat-induced alterations. Additionally, CDs can lengthen the shelf-life of food items and cover up or lessen unpleasant tastes or odors [51].

Chitosan has attracted a lot of attention for the encapsulation and delivery of bioactive substances because of its exceptional functionality. However, in the latest mentioned study, in order to increase the stability of C3G, chitosan, chitosan oligosaccharides, and carboxymethyl chitosan were combined with ionic crosslinking agents γ-polyglutamic acid nanoparticles [49]. Similarly, Ge et al. generated new nanocomplexes in their work to stabilize C3G utilizing the chitosan derivatives chitosan hydrochloride (CHC) and carboxymethyl chitosan (CMC). These two distinct water-soluble chitosan derivatives, which have significant surface positive and negative charges, respectively, are able to increase the stability of C3G via intermolecular electrostatic interactions [52,53]. Chitosan is a linear cationic polysaccharide composed of β-(1–4)-linked D-glucosamine and N-acetyl-D-glucose-amine, obtained by the deacetylation of crustacean chitin. Chitosan is a non-toxic, biodegradable, biocompatible, film-forming, antioxidant, and antibacterial natural biopolymer that is second in abundance after cellulose in terms of natural biopolymers [11,52]. Moreover, the effects of C3G alone and encapsulated in chitosan nanoparticles (Nano-C3G) in a UVB-induced acute 4 photodamage animal model were assessed in another study for the encapsulation of C3G within chitosan [54]. In another recently published study, the encapsulation of cyanidin 3-*O*-glucoside-based dyes by biocompatible PEGylated phospholipid micelles has been discussed. Polymeric micelles are now fascinating and offer potential in drug delivery systems because of their biocompatibility, ease of degradation, stability, lengthy system circulation, and controlled chemical release. PEGylated phospholipids, such as those formed of phosphatidylethanolamine (DSPE) and polyethylene glycol (PEG) polymer, may self-assemble into spherical micelles with a core-shell shape in aqueous solution. These polymeric nanocarriers have been used for the encapsulation of many substances, including hydrophobic and water-soluble drugs. In the study, the color thermodynamic properties of encapsulated C3G revealed great color stability [9]. Other discussed nanocarriers of C3G are liposomes. Liposomes are nanoscale delivery systems that may hold hydrophilic compounds in their aqueous phase and localize lipophilic drugs in their phospholipid bilayer. The structural difference model in the head, tail, and bond of lipid molecules has been used to develop various functionalized liposomes, including PEGylated long-circulation liposomes, active targeting liposomes, environmental-sensitive liposomes, and multifunctional liposomes, in order to make the common liposome more adaptable to the turbulent environment of the human and animal body [55]. As a form of nanocarriers, liposomes are advantageous due to their cell-like membrane structure, high biocompatibility, low immunogenicity, ability to preserve pharmaceuticals or active ingredients, lengthen drug half-life, lower toxicity, and increase efficacy, among other factors [55]. Simply, by shielding encapsulated components from environment variables, liposomes can increase their stability and bioavailability, making them good options for drug and food nano-delivery systems [56]. There has been a lot of research on using liposomes as protective membranes in pharmaceutical and food systems [57,58]. For instance, the encapsulation of C3G within the liposomes for improving the effectiveness and stability of C3G was studied. According to the findings, liposome nanocarriers showed their efficiency in preserving the antioxidant and anticancer activities of C3G, compared to the non-encapsulated form [59].

## 5. Nanoencapsulation Techniques of Cyanidin 3-*O*-Glucoside

The limitations of the utilization of phenolic substances, mainly anthocyanin (cyanidin 3-*O*-glucoside, etc.), need to be addressed and resolved in order to decrease their thermo/photosensitivity, and increase their solubility, bioavailability, and bioactivity [60,61,62,63,64,65].

Recently, numerous encapsulation processes encompassing physical, chemical, and physicochemical approaches have been tested and implemented in food and pharmacology research centers and industries. Based on the types of bioactive compounds and their properties, diverse encapsulation techniques are performed [66]. The steadiness, diffusion, and incorporation performances of nano/encapsulation processes are predominately based on the type of nano/microcarriers. Emulsion state, film-formation, water-solubility, and inert reactions with core samples are the fundamental properties that must be had for the nano/microcarriers in encapsulation processes [67,68]. The systematic review and meta-analysis conducted in [69] on the main encapsulation processes used for anthocyanin-loaded carrier agents were categorized into five classes, which can be ranked as spray-drying (33.33%), freeze-drying (27.08%), gelation (20.83%), lipid-based carriers (14.58%), and electrohydrodynamic methods (4.14%). Furthermore, biopolymer carriers such as maltodextrin (19.56%) and gums (15.22%) have also been mentioned as the most commonly used nano/microcarriers for encapsulation of anthocyanin, including C3G, in the selected studies. In this section, the nano/encapsulation methods used to encapsulate C3G, known as an anthocyanin compound, are described (Table 3).

### 5.1. Spray-Drying Encapsulation of Cyanidin 3-O-Glucoside

Spray-drying is considered one of the oldest and most popular processes used for the nano/encapsulation of anthocyanin’s components, including C3G, found in fruits and vegetables [70]. The data of encapsulation methods collected by some researchers showed that 31.37% of investigations were based on the spray-drying technique [69]. When compared to other encapsulation processes, spray-drying is known as an effective low-cost method, capable to obtain superior-quality dry particles using largely accessible equipment [67]. This process can be used to generate anthocyanin powder loaded with C3G with increased storage stability and favors a faster production and better control over the particle size dispersion [71]. The choice of barrier materials is fundamentally the most important step in this process, where some factors should be considered for strong protection of the core material based on its physicochemical properties [67]. For an effective bioactive compound protection, the spray-drying process is generally combined with biopolymer agents such as maltodextrin, cyclodextrin, gums, modified starch, lipids, and protein nanoparticles. Inulin and sodium alginate combined with spray-drying in the encapsulation of maqui juice showed encapsulation performances of 68.6 and 47.3%, respectively. Interestingly, the authors observed that the bio-accessibility of anthocyanin, including C3G, in the generated particles was 10% higher than in maqui juice without encapsulation [72]. Kuck et al. [73] used different types of carriers, such as gum Arabic, polydextrose, and partially hydrolyzed guar gum, by spray-drying to encapsulate Bordo grape skin extract-rich anthocyanin. The results showed that the anthocyanin-loaded C3G degradation in freeze-dried powder was more sensitive to heat than in spray-dried powder, and no difference was observed in the liberation of anthocyanin and digestion time. dos Santos et al. [74] microencapsulated blackberry pomace-rich anthocyanins through spray-drying, freeze-drying, and ionic gelation processes. The authors observed that all methods showed acceptable yield (>53%) and an encapsulation performance greater than 95%. They concluded that yogurt formulation combined with spray-drying microcapsules showed an increased bioavailability of C3G compared to the addition of microcapsules from the other two processes.

### 5.2. Freeze-Drying Encapsulation of Cyanidin 3-O-Glucoside

Anthocyanin derivatives comprising C3G are very sensitive to high-temperature and heat treatments, and thus spray-drying application is sometime detrimental to anthocyanin components. Freeze-drying is regarded as one of the best drying candidates for anthocyanin, and especially for C3G encapsulation. This drying process offers the dehydration of frozen sample mixtures of core and nano/micro-carrier agents via sublimation under vacuum and reduced temperature, resulting in great chemical structure preservation and a well-reduced risk of unwanted changes in dried samples [75]. Thanks to the low operating temperature, it is a suitable method for anthocyanin and related phenolic components’ encapsulation as they are thermosensitive materials. This process yields high-quality products with acceptable sensory properties, preserves the bio-accessibility and functionality, extends the shelf-life for bioactive compounds, and improves the thermal and color stability of anthocyanin components through diverse carrier agents [76,77]. Studies investigated by some researchers on encapsulation methods’ efficiency for anthocyanin derivatives, comparing spray-drying and freeze-drying processes, indicated that encapsulated antioxidant activity and anthocyanin constituents obtained from freeze-drying were well-preserved compared to spray-drying ones [78,79]. Souza et al. [77] encapsulated anthocyanin-rich extract concentrated in C3G obtained from jaboticaba pomace using various types of encapsulating materials, such as maltodextrin, pectin, and soy protein, by the freeze-drying process. The authors observed that the carriers were capable to efficiently protect monomeric and complex anthocyanin components higher than 76%, enhancing their stability. Sour cherry skin’s anthocyanin extract in whey protein as a carrier agent isolated by the process of freeze-drying, with an encapsulation efficiency of 70.3%, was reported in [80]. The authors noticed that the carrier agent was efficient to protect the anthocyanin components from the gastric digestion, promoting their liberation into the intestine. Although this process preserves, stabilizes, and reduces the risk of undesirable components, mainly anthocyanin, including C3G, it remains expensive and time-consuming due to the longer dehydration time compared to other encapsulation methods [81].

### 5.3. Lipid-Based Encapsulation of Cyanidin 3-O-Glucoside

Among the nano/encapsulation methods, lipid-based micro/nanocarrier techniques are also one of the most applied processes for anthocyanin derivatives’ encapsulation. Two classes of lipid-based nanoencapsulation techniques can be distinguished. Emulsions, fabricated with oil, water, and surfactants in various forms. These emulsions can be formulated using a single oil in water (O/W), or vice versa (W/O), double-oil in water (O/W/O), or water in oil in water (W/O/W) emulsions [63]. These emulsions, including Pickering emulsions, are promising lipid-based encapsulation processes for food and related industries as anthocyanin-encompassing C3G distribution systems. The emulsion droplets used to trap these bioactive compounds may be obtained in a large variety of food-grade constituents, such as polysaccharides and proteins. Recent studies on emulsions’ encapsulation indicated that 5 out of 51 cases, covering 10%, focused on establishing water in oil in water (W/O/W) emulsions for encapsulating anthocyanin derivatives. Several agents, such as guar gum and whey protein, have been reported in producing W/O/W emulsions for encapsulating anthocyanin, including C3G, in plant-based food [69]. The results of Bamba et al. [82] revealed that increasing the time of water/oil emulsion from 5 to 10 min at 10,000 rpm ensured a higher anthocyanin encapsulation performance by decreasing the droplets’ dimeter.

Another class of lipid-based nano/microcarriers are liposomes, which are mostly formulated by phospholipids, oils, and different types of organic solvents to encapsulate high-value hydrophilic and hydrophobic substances, such as C3G [58,83]. These carriers contain a central watery cavity, which is suitable for entrapment of water-soluble components such anthocyanin, whereas the lipid-soluble compounds are incorporated in the oil phase [84]. Sharif et al. [69] stated that 10% (5 out of 51) of the selected published papers on encapsulated anthocyanin, including C3G, were on liposomes. Lecithin obtained from soya was reported to be the most common phospholipid used to incorporate anthocyanin through advanced techniques, such as the supercritical carbon dioxide (SC-CO_2_) technique. For instance, anthocyanin comprising cyanidin 3-*O*-glucoside-loaded liposomes from soy lecithin phospholipid was reported to have a particle size of 159 nm, with a yield of encapsulation of 50.6%. The authors propose these carriers for their potential utilization in pharmacological, functional food, nutraceutical, and associated industries [85]. Lecithin may be obtained from other sources such as sunflower and egg yolk. However, the stability of the liposomes and the encapsulation performances may have differed based on the source of the lecithin used [86].

### 5.4. Biopolymer-Based Encapsulation of Cyanidin 3-O-Glucoside

Another widely use encapsulation technique is the ionic gelation process, which is a part of a biopolymer-based encapsulation method to fabricate micro/nanocarriers to encapsulate C3G and associated anthocyanin derivatives. Ionic nano/microgels could be developed via a combination of two distinct surface-charged biopolymers [87,88]. Biopolymer nano/microcarriers have been produced by a specific biopolymer, such as carbohydrates (chitosan, maltodextrin, β-cyclodextrin, alginates, and starch, etc.), proteins (whey and soy proteins, etc.), and plant-based hydrophilic gums [89,90]. Mar et al. [91] reported that among the nano/microgels used to encapsulate anthocyanin constituents, including C3G, alginate hydrogel beads and samples coated with chitosan were more effective in encapsulating and preserving the anthocyanin. They observed higher antioxidant potential with alginate hydrogel compared to samples with whey protein concentrate and gelatine. The authors concluded that coating the sample containing anthocyanin using alginate hydrogel beads improved its bioactivity owing to the lower deterioration of the substances including C3G during food products’ storage [62]. Thermo-reversible elastic gels and film at temperatures lower than 40 °C, in weak concentrations (1 g/100 mL), may be formed by gelatine, resulting in a sol-gel passage with a continuous increase in elasticity and viscosity. Hydrogel bead carriers with their increase viscoelasticity seem to better contribute to the anthocyanin constituents’ protection compared to whey protein concentrate and chitosan coating, and thus the complexation of biopolymers (protein-polysaccharides) to fabricated ionic hydrogel provide a good opportunity to preserve C3G and other anthocyanin derivatives [68]. The encapsulation process using biopolymer material is generally performed in association with other techniques, such as spray-drying, freeze-drying, and microwave-drying methods in order to well-encapsulate and protect the compounds and facilitate their utilization by consumers. Di Santo et al. [92] and Liu et al. [93] have developed a cyanidin 3-*O*-glucoside-encapsulated chitosan nanocarrier that presented better stability and blood compatibility, and strongly reduced UVB-induced lipid oxidation and skin deterioration. Another study in [48] focused on cyanidin 3-*O*-glucoside-encapsulated chitosan, using B-cyclodextrin-epiclorohydrin-grafted carboxymethyl chitosan (B-CD-EP-CMC) as a nanocarrier. Their results showed that this encapsulation agent could significantly enhance the stability of C3G against thermal or light degradation. The authors conclude that the findings might open a route for the production and application of novel nanoparticle materials in C3G-associated nutraceuticals.

### 5.5. Electrohydrodynamic Encapsulation of Cyanidin 3-O-Glucoside

This encapsulation process composed of electrospinning and electro-spraying is regarded as an alternative technique to conventional ones. For instance, the electrohydrodynamic process has been newly proposed as a beneficial, cost-effective, simple, and flexible technique for fabricating encapsulation materials for different bioactive constituents such as anthocyanin, mainly C3G [66,94,95]. This process is especially advantageous for the encapsulation and preservation of thermo/photosensitive molecules such as cyanidin 3-*O*-glucoside and other anthocyanin components. Different electrohydrodynamic production processes which are able to generate encapsulation barriers in one step, with low temperature, nontoxic reagents, and lower interaction with bioactive components, yielding high loading efficiencies, have been reported in previous studies [96]. The electrospinning and electro-spraying techniques are almost similar in functionality and apparatus design, and the main differences between these two processes are the final products generated, namely nanodroplets for the electro-spraying process and nanofibers for the electrospinning method. The distinction of the nanofibers and nanodroplets obtained is evaluated by the intrinsic viscosity of the solution and biomaterials’ concentration [66]. Isik et al. [97] encapsulated anthocyanin from sour cherry using gelatin or gelatin-lacto albumin as an encapsulating material via the electrospinning process. The results indicated an encapsulation efficiency of 79.2% and 70.2%, respectively, with improved stability, bio-accessibility, and protection of C3G, eight-fold better than the non-encapsulated matrix. A recent study investigated by Atay et al. [94], using the electro-spraying process to encapsulate anthocyanin-rich black carrot obtained with a mixed solution of chitosan/gelatin, showed successful results with an encapsulation efficiency of 76%, with increased stability and protection of C3G and other anthocyanin components. Although this process provides numerous advantages, it is a slow process and generates low yields. These challenges restrict its commercial exploitation and large-scale application [98].

## 6. Bioavailability and Bio-Accessibility of Cyanidin 3-*O*-Glucoside

**Figure 3:**
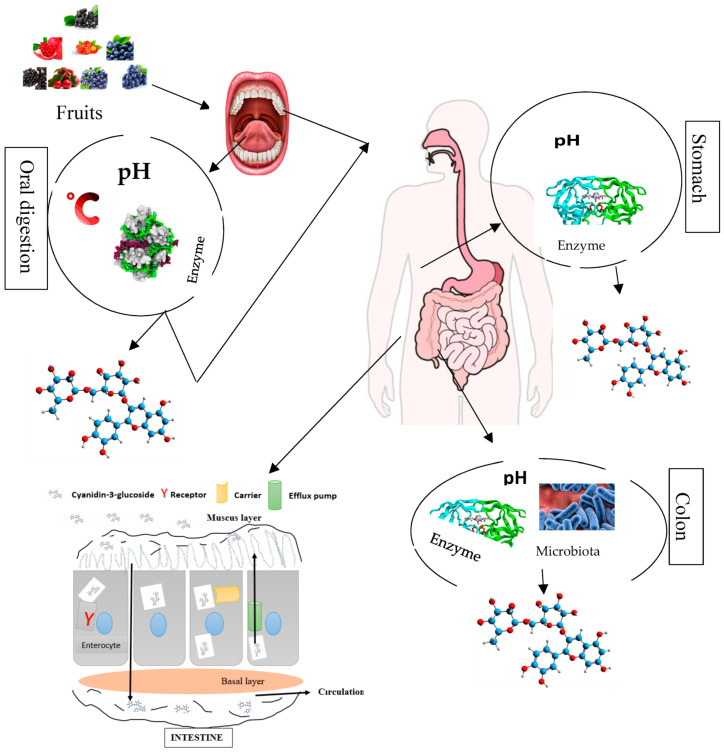
Overview of anthocyanin (cyanidin 3-*O*-glucoside) bio-transformations (degradation and absorption) in different compartments of the human gastrointestinal–digestive system.

The bio-accessibility of anthocyanin components comprising C3G through the different compartments of the digestive tract firstly start from their release from the food matrix. C3G and other anthocyanin components after liberation enter in contact with some micro-molecules, enzymes, and other phenolic compounds, which might affect their bio-accessibility [99,100,101,102,103]. Anthocyanin components including C3G have frequently been reported to poorly assimilate in the different compartments of the digestive tract or gastrointestinal track, and they have a low diffusion rate, which limits their application in functional food (foods with physiological benefits). The effect of anthocyanin constituents’, especially cyanidin 3-*O*-glucoside, health properties might largely depend on their bioavailability, encompassing bio-accessibility, absorption, diffusion, metabolism, distribution, and excretion (Figure 3). The thermo/photosensitivity, instability, the neutral or basic pH sensitivity, oxygen, enzymes, metal ions, and organic acids cause C3G to rapidly degrade [104]. For instance, the authors of [6] stated that C3G experience substantial metabolism through deglycosylation, sulfation, and glucuronidation, resulting in a low level of diffusion and bio-accessibility. The authors affirmed that after an in vitro gastrointestinal digestion simulation, less than 40% of unaltered C3G was recovered. Another study by Peixoto et al. [105] demonstrated that approximately 10 to 45% of anthocyanin comprising C3G after a gastrointestinal digestion assay was bio-accessible. Moreover, the authors stated that the high polarity of C3G decreases its absorption via intestinal epithelia. Czank et al. [106] assessed the bioavailability of C3G on human volunteers through ^13^C-labeled cyanidin 3-*O*-glucoside. The authors reported that the mean relative bioavailability was 12.38% based on the excretion of ^13^C via urine and breath, also observing a metabolite quantity approximately 42 times greater than those of the parent anthocyanin. Furthermore, nano/microcarriers have been reported to decrease the bio-accessibility of anthocyanins, while some have been confirmed to improve to bio-accessibility and bioavailability of C3G.

For example, the addition of pectin as an encapsulating material considerably decreased the bioavailability of C3G to about 14% after in vitro digestion [107]. Zou et al. [5] reported an increased bio-accessibility of C3G after the addition of low-viscosity alginate during simulated gastrointestinal digestion. Interestingly, the authors also observed a significant enhancement in the bio-accessibility, by 27.4%, of C3G-LVA in mouse plasma. They concluded that low-viscosity alginate could be a powerful encapsulating material and could be used as an oral carrier for dietary anthocyanin. Shishir et al. [11] evaluated the bio-accessibility of C3G through a specific colon carrier fabricated from polysaccharide and nanofibersolosome during simulated gastrointestinal digestion. A simulated in vitro gastrointestinal digestion and in vivo (rat) biotransformation study conducted by Chen et al. [108] showed that after gastrointestinal digestion, C3G was identified with a recovery rate of 88.31% in the gastric-digestion section. The authors also revealed that C3G was degraded into protocatechuic acid and 2,4,6-trihydroxybenzaldehyde, leading to rapid absorption and distribution in tissues and the brain.

## 7. Storage, Thermal, pH, and Light Stabilities of the Encapsulated Cyanidin 3-*O*-Glucoside

By monitoring the changes in color and the anthocyanin concentration during the accelerated tests, the stability of natural anthocyanin pigments may be assessed [45]. However, C3G is chemically unstable. The phenolic hydroxyl groups in the structure of C3G prevent it from steadily existing for a very long time [109]. C3G degradation was likely caused by the hydrolysis of the carbon atom at position C2, which caused the pyrylium ring of anthocyanin to open and generate a chalcone structure [45]. The stability of C3G is influenced by a number of variables. It degrades rapidly during food processing when exposed to neutral or basic pH values, elevated temperatures, oxygen, enzymes, light, pressure, and other reactive substances, such as ascorbic acid and metallic ions [6,49,110]. However, C3G and other compounds that are susceptible to heat and light are stabilized and given a longer shelf-life through microencapsulation [101]. This technology is quickly evolving and offers a number of advantages, including high specialization and low cost [111].

### 7.1. Storage Stability

As discussed earlier, C3G is easily impacted by a variety of reactions happening in food products. The biggest concern with the preservation of C3G is their instability caused by temperature, oxygen, light, and certain enzymes during the storage of the product. According to the evidence, C3G liposomes produced using the pH gradient loading approach have much better stability (85.4%), antioxidant activity, and skin permeability and may remain stable for 14 days in vitro under physiological circumstances [112]. Briefly, C3G in RAH liposomes are protected from degradation during storage and retain antioxidant activity without cytotoxicity [112]. According to other studies, black carrot-encapsulated C3G liposomes remained stable after three weeks of storage [58].

### 7.2. Thermal Stability

It is well-known that C3G is a notoriously unstable plant pigment that may deteriorate very quickly when exposed to heat. According to some theories, the rapid decomposition of anthocyanin at higher temperatures may be caused by the hydrolysis of the 3-glycoside structure, which acts as a barrier for unstable anthocyanin. The other hypothesis is that the hydrolysis of the pyrilium ring produced chalcone, which is what gives foods containing anthocyanin their brown color [113]. However, the encapsulation of C3G within the ferritin cage demonstrated great thermal stability upon examination by UV-vis spectrophotometry in combination with HPLC, using free anthocyanin as a reference. When compared to free C3G, the thermal and photo-stabilities of the protein nanocage-enclosed C3G were significantly increased. This results from the contact between C3G molecules and amino acid residues on the inner surface of apoferritin, which protects the molecules of C3G within it [44]. Qin et al. [43] showed that the encapsulation of C3G with whey protein isolate-glucose developed a novel system that is stable under acidic and high-temperature conditions. According to the results, the loading of C3G into protein particles successfully improved the thermal stability of C3G under different pH conditions [43]. However, the thermal stability of anthocyanin varies with temperature and pH [113]. C3G are only thermally stable at pH < 3. Thereby, the challenge in using C3G in industrial settings is improving their stability at pH ≥ 3 during processing and storage.

In one published study, the thermal stability of the encapsulated C3G was studied at 4 °C, room temperature, 45 °C, and 60 °C. The degradation of the encapsulated C3G was slower than that of the non-encapsulated one at all temperatures, with the exception of 60 °C. Interestingly, at 60 °C, the encapsulated C3G degraded more quickly than the non-encapsulated form of C3G, however the results could be explained due to the degradation of the encapsulation materials at this temperature [114]. In another study, the encapsulated form of C3G had a high retention rate of 83.34% when placed in the dark at 4 °C for 28 days, compared to 76.63% for the non-encapsulated form. The retention rate was 66.80% for the encapsulated form and only 48.53% for the non-encapsulated form in the same dark circumstances and increased temperature of 25 °C [57]. Sari et al. found that C3G breakdown was increased by heating, which led to a drop in color intensity and the creation of a polymeric color. The C3G were more sensitive to degrading at 98 °C than at 80 °C, whether in their natural or co-pigmented forms. Additionally, the natural C3G in the beverage model demonstrated stronger stability than the co-pigmented one at the two heating temperatures of 80 and 98 °C [115]. The C3G-loaded chitosan hydrochloride and carboxymethyl chitosan nanocomplexes (encapsulated form) have performed thermal stability tests at three different conventional temperatures: 4 °C (refrigeration temperature), 25 °C (room temperature), and 40 °C (accelerated temperature storage conditions). Samples were stored in the dark at each of these three temperatures. The thermal stability of the chitosan-encapsulated samples at various storage temperatures was found to be higher than that of the unencapsulated samples, according to the results in [53]. The same result has been reported when C3G was encapsulated with casein protein. Comparing the casein-encapsulated C3G solution to the unencapsulated C3G solution following heat treatment, the encapsulated form revealed a desired protective effect [45]. The literature, however, suggests that there are two approaches to explain this increase in stability. First, the electrostatic interactions between oppositely charged chitosan derivatives, where nano-C3G increased the thermal stability of nanocomplexes. The hydration of C3G might be prevented by the nanocomplex formation through ionic interactions between the chitosan derivatives and the C3G, which would retain more stability [53]. Second, the thermal stability of anthocyanin was greatly enhanced by water-soluble carbohydrates, according to a recent study, by lowering the water activity around them [116].

### 7.3. Light Stability

Light is another factor that affects the color stability of C3G. The effect of light on encapsulated and non-encapsulated C3G within maltodextrin and chitosan was investigated. The encapsulated powders were stored in dark and light conditions for 15 days, and then their light stability was assessed using UV and IR spectroscopic methods. Data showed that maltodextrin and chitosan can increase the light stability of C3G because the encapsulated polymeric matrices protect the C3G by inhibiting oxidation, which may explain why non-encapsulated C3G degraded more quickly than encapsulated C3G [114]. In comparison to non-encapsulated C3G samples, the half-life of all encapsulated C3G samples increased during storage [117]. In another study, samples of the encapsulated C3G form were exposed to the light source for 28 days at 25 °C while they were incubated in a biochemical incubator with a white fluorescent light. According to the results, the retention rate for the encapsulated form after storage at 25 °C under white fluorescent light was 43.38%, whereas it was only 28.09% for the non-encapsulated form. Generally, the breakdown of C3G was increased by the presence of white fluorescent light, but the encapsulation process significantly reduced this impact [57]. Ouyang et al. demonstrated an improvement in the thermal, oxidation, light (30 h), and storage stability of casein-encapsulated C3G compared to the non-encapsulated C3G [45].

### 7.4. pH Sensitivity

The pH is a significant additional factor that affects the stability of C3G. Anthocyanin nanoparticles are pH-sensitive in vitro, as demonstrated by Sun et al.’s research, which showed that, in comparison to pH 6.8 and pH 7.4, C3G encapsulated with chitosan nanoparticles at pH 5.3 demonstrated the greatest release ratio [49]. According to the observation of a few in vitro studies, at a pH of around 3 or lower, the anthocyanin is orange or red and exists as a flavylium cation [113]. The hydration process of the flavylium cation and the proton transfer reactions linked to the acidic hydroxyl groups of the aglycone compete kinetically and thermodynamically when the pH is raised. While the initial reaction produces an inert carbinol pseudo-base that can undergo ring opening to become a chalcone pseudo-base, the second reaction can produce quinonoidal bases at pH levels between 6 and 7 with the generation of purple, resonance-stabilized quinonoid anions [113]. As a result of pH-dependent equilibrium forms, they may exhibit different colors in aqueous solutions: at very acidic pH, anthocyanin are primarily in their red cationic form (AH+), but as pH increases, the flavylium cation immediately undergoes deprotonation to give rise to quinoidal bases (A), and at the same time, but more slowly, it may hydrate to form colorless hemiketal species (B2), which then quickly tautomerize to yield cis-chalcone. The species in question might then isomerize into trans-chalcone [9]. However, the hydration on the 2-position of the anthocyanidin structure may be the cause of the color stability deterioration in acidic environments [43]. In one study, the pH stability of the encapsulated C3G with chitosan has been evaluated by determining the retention amount between the encapsulated and non-encapsulated C3G. The nanoparticles were placed for 6 days at different pH conditions ranging from 2.0 to 6.0. Data showed that the remaining amount of C3G (%) in the encapsulated samples was much higher than their counterparts at all the same pH values [53]. In higher pH circumstances, C3G was unstable, and its rate of degradation accelerated. C3G was stable at lower pH (2–3) levels. It has been declared that after the encapsulation process, C3G is better protected against pH-related degradation [53,118].

## 8. Uses of the Encapsulated Cyanidin 3-*O*-Glucoside

Numerous studies have demonstrated that C3G-encapsulated nanoparticles have strong encapsulation efficiency as well as the potential to further enhance and provide a framework for the investigation of C3G nanoparticle applications in functional foods [109,119,120]. Converging research has recently shown that the natural pigment C3G has an impact on several physiological processes, including inflammation, cardiovascular disease, cancer, antioxidant activity, and antidiabetic effects [121]. C3G is widely used as a food additive because of its appealing hue and advantageous bioactivity, which enhances both food color and health functions [53]. C3G could possess the ability to scavenge free radicals, which might stop the oxidation of low-density lipoproteins and have a good impact on obesity, inflammation, and chronic gut inflammatory disorders [59]. According to a number of studies, C3G prevents the negative effects of UV-B radiation, regulates important components of carcinogenesis, stops cancer cells from proliferating, and triggers the death of cancer cells, decreases oxidative stress, prevents oxidative stress brought on by H_2_O_2_ in human embryonic kidney (HEK 293) cells, triggers cell death, and prevents cell migration in TNF-α-challenged RASMCs in vitro [59]. According to Sivasinprasasn, C3G pre-treatment suppresses the NF-kB signaling pathway [119] (Table 4).

C3G was shown to inhibit UVB-induced apoptosis in human HaCaT keratinocytes [54]. In vivo, C3G can guard against UVB-induced epidermal deterioration. However, the therapeutic use of C3G and its industrial applications as functional food components has been constrained by how quickly it degrades. Nowadays, polymeric nanoparticles are currently playing a crucial role in the advancement of therapeutic and calleidic systems in the new wave of the development of cosmetic or pharmaceutical dosage forms due to their capacity for regulating drug release and enhancing the stability of pharmaceuticals [54]. The nano-C3G encapsulation within chitosan according to the animal experiment showed that nano-C3G could efficiently lower the levels of lipid peroxidation, malondialdehyde, and 8-hydroxy-2′-deoxyguanosine caused by UVB exposure, as well as downregulate the expression of p53, Bcl-2-associated X (Bax), caspase-3 and -9, and balance the B-cell lymphoma-2/leukemia-2 ratio [54]. Liang et al. investigated the effect of C3G encapsulated with liposomes on normal GES-1 cells by evaluating cell viability and mitochondrial structure. The primary cell line in the human stomach is the human gastric epithelial cell line (GES-1 cells), and the health of the stomach is crucial for body health since it is the primary organ through which many biochemical interactions occur [111]. According to the findings, C3G liposomes had no impact on the shape and structure of mitochondria, and at the same time, it can increase the antioxidant activity and stability of C3G. In a recently published study, the anti-inflammatory and anti-apoptotic activities of the encapsulated C3G liposomes have been evaluated in vitro [109]. According to evidence, C3G liposomes are effective in inhibiting several inflammatory factors, such as tumor necrosis factor-a (TNF-a), interleukin (I L)-1β, IL-6, and IL-8, stimulated by lipopolysaccharide. Furthermore, C3G liposomes might have the potential for protecting macrophages from apoptosis. However, it has been concluded that liposome encapsulation provides C3G with some level of protection [109]. Regarding the application of C3G in food processing, as an alternative to synthetic colors for making jelly powder, the C3G was encapsulated using several encapsulating materials, including gum Arabic, maltodextrin, gelatin, or their combination [117]. In another recently published study, the encapsulated C3G extracted from *Solanum melongena* L. bark has been used as a natural dye in yogurts [120]. Additionally, encapsulated C3G-bovine serum albumin nanoparticles demonstrated a promising approach for the enhancement of C3G stability in the beverage industry [46].

## 9. Conclusions

Anthocyanin C3G is a secondary metabolite naturally present in herbal origin sources. It exerts tremendous health benefits, including regulation of cholesterol, antioxidant, anti-inflammatory, hepatoprotective, anticancer, anti-obesity, and antidiabetic effects. The recovery of C3G is influenced by many factors, such as extraction techniques, extraction conditions, and raw materials. Likewise, C3G is sensitive to many factors and its stability is affected by pH, light, temperature, and storage time. The nanoencapsulation improves the stability and bioavailability of C3G. Most nanocarriers used for its nanoencapsulation are proteins, polysaccharides, polymeric micelles, and phospholipid-based materials. Furthermore, the encapsulation techniques are also determinant factors that influence the production yield and availability of C3G. C3G is used as a food additive and colorant, as well as for therapeutic purposes.

## Figures and Tables

**Table 1:** Heath benefits of cyanidin 3-*O*-glucoside.

Compounds	Biological Activities	References
Cyanidin 3-*O*-glucoside and its phenolic acid metabolites (protocatechuic acid and ferulic acid)	Regulation of cholesterol and anti-inflammatory effect	[14]
Cyanidin 3-*O*-β-glucoside chloride and cyanidin chloride	Blocking accumulation of cholesterol and anti-inflammatory effect	[15]
Cyanidin 3-*O*-glucoside and peonidin-3-glucoside	Anticancer	[16]
Cyanidin 3-*O*-glucoside	Antidiabetic effect	[17]
Cyanidin 3-*O*-glucoside	Antioxidant and cardioprotective effects	[18]
Binding of pectin and cyanidin 3-*O*-glucoside	Antioxidant activity	[19]
Cyanidin 3-*O*-glucoside	Liver-protective effects	[5]
Cyanidin 3-*O*-glucoside	Anti-obesity	[6]
Cyanidin 3-*O*-glucoside	Hepatoprotective effect	[7,8]
Cyanidin 3-*O*-glucoside	Glutathione depletion, lipid peroxidation, and myeloperoxidase inhibition	[9]
Quercetin and cyanidin 3-*O*-glucoside	Hypolipidemic and antioxidant effects	[10]
Cyanidin 3-*O*-glucoside	Reversion of cardiovascular, liver, and metabolic signs	[11]

**Table 2:** Recovery of cyanidin 3-*O*-glucoside by different extraction techniques.

Extraction Methods	Type of Solvents	Amounts of C3G	Raw Materials	References
Maceration	Ethanol	0.07 ± 0.01–0.14 ± 0.02 mg/g	Chokeberry	[12]
	Ethanol	21.76 ± 0.18	Black currant	[13]
Soxhlet extraction	Ethanol, water, acidified mixture of ethanol + water, and acidified water	0.12 ± 0.01 mg/g	Juçara	[14]
Orbital shaking technique	0.5% trifluoroacetic acid in methanol	0.21%	Sandalwood	[15]
Agitated bed extraction	Ethanol, water, acidified mixture of ethanol + water, and acidified water	0.18 ± 0.02 mg/g	Juçara	[14]
Ultrasound-assisted extraction	80% methanol + 1% HCl	0.90 ± 0.04 mg/100 g	Sour cherry	[16]
	Ethanol, water, acidified mixture of ethanol + water or acidified water	0.174 ± 0.002 mg/g	Juçara	[14]
	Water, ethanol, methanol, and deep eutectic solvents	114.5 ± 0.87–1768.57 ± 4.34 mg/L	Blackberry	[17]
	20, 60, and 100% methanol	25.5 ± 0.30	Raspberries	[18]
	Ethanol	4.9 mg/g	Jabuticaba peel	[19]
	Water, ethanol, methanol, and deep eutectic solvents	55.01 ± 0.01–114.24 ± 0.06	Sumac	[20]
Microwave-assisted extraction	80% methanol + 1% HCl	1.65 ± 0.09 mg/100 g	Sour cherry	[16]
High hydrostatic pressure	80% methanol + 1% HCl	0.46 ± 0.13 mg/100 g	Sour cherry	[16]
Homogenate-assisted extraction	Water, ethanol, methanol, and deep eutectic solvents	40.50 ± 0.04–112.15 ± 0.02 mg/L	Sumac	[20]
Pressurized fluid extraction	98% methanol + 2% HCl	0.23 ± 0.00–0.46 ± 0.00 mg/g	Cranberry pomace	[21]
	Ethanol, water, acidified mixture of ethanol + water, and acidified water	0.003 ± 0.0002–0.099 ± 0.007 mg/g	Juçara	[14]
Supercritical CO_2_ extraction	Co-solvents (Water or ethanol)	62.0–69.6%	Purple corn cob	[22]

**Table 3:** Characteristics of different nano/microencapsulation processes used to protect cyanidin 3-*O*-glucoside.

Products	Anthocyanins	Carrier Agents	Encapsulation	References
Rose residue	Cyanidin 3-*O*-glucoside	Gum Arabic, Maltodextrin	Spray-drying, Freeze-drying	[23]
Red cabbage	Cyanidin 3-*O*-glucoside	Maltodextrin	Spray-drying	[24]
Black rice	Cyanidin 3-*O*-glucoside	Maltodextrin,	Spray-drying	[25]
Barberry extract	Cyanidin 3-*O*-glucoside	Gum Arabic Maltodextrin	Spray-drying	[26]
Blueberry extract	Cyanidin 3-*O*-glucoside	Pectin, Maltodextrin	Spray-drying	[27]
Black chokeberry	Cyanidin 3-*O*-glucoside	Pectin, guar gum, gum Arabic Maltodextrin	Spray-drying	[28]
Bilberry extract	Cyanidin 3-*O*-glucoside	Pectin	Spray-drying	[29]
Black rice	Cyanidin 3-*O*-glucoside	Whey protein Maltodextrin	Spray-drying	[30]
Mulberry juice	Cyanidin 3-*O*-glucoside	Whey protein Maltodextrin	Spray-drying	[31]
Blueberry	Cyanidin 3-*O*-glucoside	Beeswax, GMO	Emulsification	[32]
*Z. mays* extract	Cyanidin 3-*O*-glucoside	Span 60, cholesterol	Emulsification	[33]
Bilberry extract	Cyanidin 3-*O*-glucoside	Whey protein isolate (Bulk hydrogels)	Thermogelation	[34]
Bilberry extract	Cyanidin 3-*O*-glucoside	Whey protein isolated (microgels)	Thermogelation, Emulsification	[35]
Black carrot	Cyanidin 3-*O*-glucoside	Whey protein isolate (microgels)	Thermogelation, emulsification	[36]
	Cyanidin 3-*O*-glucoside	Whey protein isolate-glucose (nanogels)	Thermogelation, Maillard reaction	[37]
	Cyanidin 3-*O*-glucoside	ovalbumin-dextran (nanogels)	Thermogelation, Maillard reaction	[38]
Purple corn	Cyanidin 3-*O*-glucoside	Pectin, alginate, sodium citrate (Hydrogels beads)	Ionic gelation	[39]
Blueberry extract	Cyanidin 3-*O*-glucoside	Pectin, alginate, sodium citrate (Hydrogels beads)	Ionic gelation	[39]
Purple rice bran	Cyanidin 3-*O*-glucoside	Pectin, Ca^2+^, zein (hydrogel beads)	Ionic gelation, complexation	[40]
Black soybean	Cyanidin 3-*O*-glucoside	Chitosan (nanoparticles)	Ionic gelation	[41]
Black rice	Cyanidin 3-*O*-glucoside	Chitosan chondoitin sulfate	Polyelectrolyte complexation	[42]
Elderberry	Cyanidin 3-*O*-glucoside	Chitosan chondoitin sulfate	Polyelectrolyte complexation	[43]
Blueberry	Cyanidin 3-*O*-glucoside	Chondroitin sulfate kappa-carrageenan	Polyelectrolyte complexation	[44]
Blueberry	Cyanidin 3-*O*-glucoside	Chondroitin sulfate, chitosan, alginate	Ionic gelation	[43]
Chokeberry	Cyanidin 3-*O*-glucoside	Maltodextrin guar gum	Spray-drying	[28]
Blueberry	Cyanidin 3-*O*-glucoside	Maltodextrin DE20 hi-maize, inulin, gum Arabic	Spray-drying	[45]
Mulberry	Cyanidin 3-*O*-glucoside	Alginate/chitosan	Spray-drying, external gelation	[46]
Pomegranate	Cyanidin 3-*O*-glucoside	Maltodextrin (5%, 10%, 15%)	Freeze-drying	[47]
Pitanga	Cyanidin 3-*O*-glucoside	Fructans	Spray-drying	[48]
Saffron	Cyanidin 3-*O*-glucoside	B-glucan, B-cyclodextrin	Spray-drying	[49]
Black rice	Cyanidin 3-*O*-glucoside	Maltodextrin, gum Arabic, whey protein	Spray-drying, Ionic gelation	[30]
Hibiscus	Cyanidin 3-*O*-glucoside	Pectin	Ionic gelation	[50]
Sour cherry concentrate	Cyanidin 3-*O*-glucoside	Gelatin/gelatin-lactalbumin	Electrospinning	[51]
Black carrot extract	cyanidin 3-*O*-glucoside	Chitosan/gelatin	Electro-spraying	[52]
Bilberry extract	Cyanidin 3-*O*-glucoside	Soy lecithin	Liposome	[53]

**Table 4:** Summarization of the health benefits and potential applications of C3G in food processing.

Field of Use	Advantages	References
Health benefits	- Has a positive impact on inflammation and cardiovascular disease. - Has anticancer and antioxidant activity. - Antidiabetic effects. - C3G could possess the ability to scavenge free radicals. - Has a good impact on obesity, inflammation, and chronic gut inflammatory disorders. - C3G prevents the negative effects of UV-B radiation. - Stops cancer cells from proliferating. - Has the potential for protecting macrophages from apoptosis.	[59] [119] [109] [121]
Application of C3G in food processing	- Alternative to synthetic color for making jelly powder. - As a natural dye in yogurts.	[117] [120]

## Data Availability

Not applicable.
